# Conventional Hypoglycaemic Agents and the Risk of Lung Cancer in Patients with Diabetes: A Meta-Analysis

**DOI:** 10.1371/journal.pone.0099577

**Published:** 2014-06-12

**Authors:** Ying Wu, Hong-Bing Liu, Xue-Fei Shi, Yong Song

**Affiliations:** Department of Respiratory Medicine, Jinling Hospital, Nanjing University School of Medicine, Nanjing, China; University of Milan, Italy

## Abstract

**Background:**

Accumulating evidence suggests that hypoglycaemic agents influence lung cancer risk in patients with diabetes. It remains to be fully elucidated whether conventional hypoglycaemic agents (metformin, sulfonylureas, thiazolidinediones [TZDs] or insulin) affect lung cancer incidence in patients with diabetes.

**Methods:**

We performed a meta-analysis using EMBASE, MEDLINE and Web of Science to search randomised controlled trials (RCTs), cohort studies, and case-control studies published up to October 2013 that assessed the effects of metformin, sulfonylurea, TZDs or insulin on lung cancer risk in subjects with diabetes. Fixed and random effects meta-analysis models were used, and the effect size was expressed as a summary odds ratio (OR) with 95% confidence intervals (CI). The Grades of Research, Assessment, Development and Evaluation (GRADE) approach was applied to define the quality of the evidence.

**Results:**

Analysis of 15 studies (11 cohort studies, 2 case-control studies, and 2 RCTs) showed that metformin use was associated with a 15% reduction in risk of lung cancer (OR 0.85, 95% CI 0.77 to 0.92), but this finding was not supported by sub-analysis of smoking-adjusted studies (OR 0.84, 95% CI 0.61 to 1.06). Moreover, sulfonylurea or TZDs use was not associated with increased or decreased lung cancer risk, respectively (OR 1.10, 95% CI 0.93 to 1.26), (OR 0.86, 95% CI 0.70 to 1.02). Higher lung cancer risk was related to insulin (OR 1.23, 95% CI 1.10 to 1.35). However, all data from RCTs failed to demonstrate a statistically significant effect.

**Conclusions:**

This analysis demonstrated that metformin use may reduce lung cancer risk in patients with diabetes but not in a smoking-adjusted subgroup and that insulin use may be associated with an increased lung cancer risk in subjects with diabetes.

## Introduction

Lung cancer is the leading cause of cancer-related death both in the USA and around the world [1]. Diabetes is a rising common problem in many countries worldwide [2]. Diabetes has been established as an independent risk factor for lung cancer [3].Increasing evidence has shown that conventional glucose-lowering drugs such as insulin, insulin sensitisers and secretagogues, may influence the risk of cancer. Metformin exerts an anticancer effect by both insulin-dependent and insulin-independent mechanisms [4]. Thiazolidinediones (TZDs), synthetic peroxisome proliferator-activated receptor gamma (PPARγ) ligands, suppress cancer cell proliferation through the interplay between apoptosis and autophagy [5] [6] [7]. However, sulfonylureas, as insulin secretagogues, can promote cell proliferation and seem to have oncogenic effects [8]. Several observational studies have suggested that the use of metformin and TZDs is associated with a decreased risk of lung cancer compared with other glucose-lowering drugs [9] [10] [11]. In contrast, others have shown a non-significant protective effect on lung cancer [12] [13]. Likewise, insulin and insulin secretagogues have been shown to be related to higher lung cancer incidence and cancer-related mortality [14] [15]. But others have shown no harmful or even protective effects [16] [17]. Although there have been some systematic reviews on the relevant subject, some results of previous systematic reviews remain inconsistent [18], [19]. Some other earlier systematic reviews do not specialize in lung cancer or are limited by small study sizes [20] [21].

To investigate the relationship between the use of glucose-lowering drug (metformin, TZDs, sulfonylureas, and insulin) and lung cancer risk in patients with diabetes, we conducted a meta-analysis of existing randomised controlled trials (RCTs) and observational studies.

## Materials and Methods

### Literature search

We carried out a computerised search of published research studies in the Medline, Embase and Web of Science databases by using the following search terms: “metformin OR thiazolidinediones OR insulin therapy OR sulfonylurea compounds OR hypoglycemic agents” AND “diabetes” AND “neoplasms” combined with “risk”. An upper publication date limit of October, 2013 was used, but no lower date limit was applied. All English language publications were considered.

### Selection criteria

All potentially relevant studies were retrieved and assessed for inclusion according to the following criteria: (1) study must have evaluated lung cancer risk in patients with diabetes on the basis of type of hypoglycaemic agent (metformin, sulfonylureas, rosiglitazone, pioglitazone, insulin); (2) study design must have been RCT, case–control or cohort; (3) study must have reported the hazard ratio (HR) or odds ratio (OR); and (4) population consisted of adult patients. The observational studies not adjusted for any confounder or duplicate publications of studies in the same population were excluded. When the same patient population appeared in several publications, only the most recent or comprehensive study was selected. Disagreements were resolved by discussion.

### Data extraction and assessment of quality

Data was extracted from all selected studies by two reviewers working independently, using a standardised form to ensure capture of all relevant information. The following data were collected from each study: first author's name, publication date, country, study design (case-control, cohort, or RCT), time period, mean follow-up time, outcome assessment, type of diabetes, total subjects, lung cancer cases, ratio of each glucose-lowering drug. Where available, adjusted OR or HR values were analysed. If data from any of the above categories were not reported in the primary study, items were treated as ‘‘not available’’. For all analyses, the control group was composed of patients with diabetes not exposed to medication of interest. We did not require a minimum number of patients for a study to be included in our meta-analysis.

Two reviewers assessed the risk of bias in observational studies according to the Newcastle – Ottawa Scale which included selection, comparability of studies groups, and ascertainment of exposure or outcome [22]. The overall maximum score was 9 points. The two reviewers applied the Cochrane Collaboration' s tool to evaluate the risk of bias of randomised trials [23]. This tool based on randomisation, blinding allocation concealment procedures and loss to follow up. Disagreements were resolved by discussion and consensus.

### Risk of bias across studies

The Grades of Research, Assessment, Development and Evaluation (GRADE) framework was used to determine quality of evidence for each meta-analysis. Each meta-analysis could receive a recommendation with four levels of evidence quality, ranging from very low to high [24]. Meta-analysis of RCTs was graded as high quality, however they could be downgraded because of factors such as design limitations, indirectness, inconsistency, imprecision and publication bias. Evidences from observational studies were classified as low quality by default, but they also could be downgraded by the factors as above or upgraded due to large magnitude of effect, potential confounding and dose-response relationship.

### Statistical analysis

This meta-analysis was conducted according to Cochrane Handbook and PRISMA guidelines (Checklist S1) [25] [26]. Adjusted OR or HR with 95% confidence intervals (CIs) was calculated to determine the assessment of risk of lung cancer in patients with diabetes on the basis of the type of glucose-lowering drug. Since the frequency was relatively low (less than 1%) [27], the OR in case-control studies was considered approximations of HR in cohort studies [28]. OR or HR indicated statistically significant at the p<0.05 level if the 95% CI did not include the value one. The heterogeneity across the included studies was assessed using the chi squared (χ^2^)-based Q test [29]. A *P* value less than 0.1 for the Q test indicated a lack of heterogeneity between studies, and indicated that the pooled OR estimate of each study should be calculated by the Mantel–Haenszel fixed-effects model. Otherwise, the random-effects model was used [30], [31]. Subgroup analyses were then carried out by study design, location and whether the study was adjusted for smoking or other glucose-lowering drugs. Due to significant differences in the design of observational studies and post-hoc analysis of RCTs, data from these RCTs were analysed and presented separately. Potential publication bias was estimated by the funnel plot, in which the standard error of log (OR) of each study was plotted against its log (OR). An asymmetric plot suggested a possible publication bias. Funnel plot asymmetry was assessed by Egger's linear regression test. The significance of the intercept was determined by the t test, as suggested by Egger and Smith [32] (P<0.05 was representative of a statistically significant publication bias). All calculations were carried out with the STATA version 12.0 statistical software package (Stata Corp., College Station, TX, USA).

This was a literature-based study, and ethics approval was not required.

## Results

### Flow of included studies

**Figure 1:**
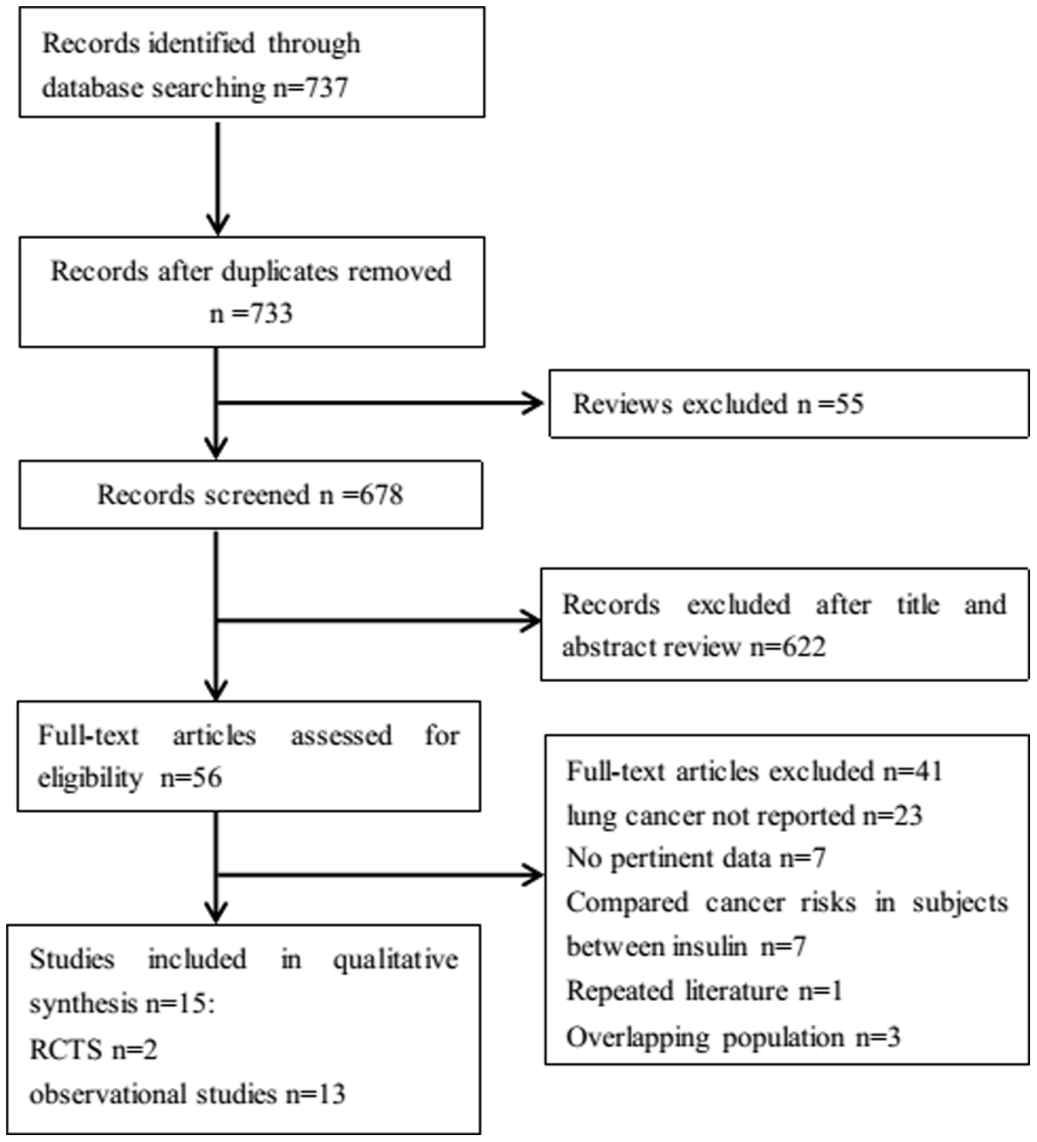
Flow chart demonstrating the progression of the trials in the review. The flowchart of selecting procedure and the exclusive reason of studies are summarized.

A total of 18 potentially relevant studies were identified by the initial computerised search. There were five Taiwanese studies from the same cohort, and hence, one of these was included in the analysis for metformin, sulfonylureas and insulin, while another was analysed for TZDs. Two studies were from the UK-based General Practice Research Database, one of which was analysed for metformin and the other for sulfonylureas and insulin. Hence, the remaining 15 studies fulfilled the inclusion criteria and were included in meta-analysis [9], [10], [11], [12], [13], [14], [15], [16], [17], [27], [33], [34], [35], [36]. There were 11 cohort studies, 2 case-control studies, and 2 RCTs, which were included in one publication (Figure 1).

### Study characteristics

Fifteen published studies reporting 21,089 cases of lung cancer in 2,072,425 patients with diabetes met the inclusion criteria and were ultimately analysed. Table 1 presents the main characteristics of these studies. All the patients in the included studies were on multiple glucose-lowering drugs for management of diabetes, and the comparators for the estimation of OR were determined on exposure to drug of interest and non-exposure to the same glucose-lowering drug. Most studies adjusted for the following confounders: age, sex, ethnicity, smoking, chronic lung disease, body mass index (BMI), glycosylated hemoglobin (HbA1C), diabetes duration, and other glucose-lowering drugs (Table 2). The quality of all the studies included was moderate to high. Table S1 showed the risk of bias assessment in RCTs in more detail.

**Table 1:** Characteristics of included studies assessing glucose-lowering drugs and lung cancer risk in patients with diabetes.

Author	Year	Country	Design	Time period	Mean follow-up(years)	Outcome Measures	Type of DM	Study of quality	Total subjects	Lung cancer cases	Metformin (%)	TZDs (%)	Sulfonylurea (%)	Insulin (%)
Smiechowski	2013	UK	Cohort	1988–2009	5.6	diagnostic codes	2	8 of 9	8572	808	77	NA	NA	NA
Ruiter	2012	Netherlands	Cohort	1998–2008	2.8 for metformin; 4.6 for sulfonylurea	ICD-9	NA	7 of 9	85,289	454	61.80	NA	38.20	NA
Mazzone	2012	USA	case-control	2001–2011	NA	direct review of medical record	NA	6 of 9	1014	507	27.70	8.50	NA	NA
Luo	2012	US	Cohort	2005–2010	11	ICD-O	NA	7 of 9	8,154	108	11.40	NA	NA	17.20
Lai	2012	Taiwan	Cohort	2000–2008	NA	ICD-9	NA	8 of 9	19,624	129	84.70	20.10	83.50	65.90
Bodmer	2012	U.K.	case-control	1995–2009	NA	Diagnostic codes	NA	7 of 9	7203	1029	45.80	NA	48.30	15.60
Ferrara	2011	U.S.A.	Cohort	1997–2005	2.5 for pioglitazone 3.7 for never used	KPNC Cancer Registry	NA	8 of 9	252,467	1637	45.30	12.60	52.80	20.40
Libby	2009	Scotland,	Cohort	1994–2003	NA	ICD-9 ICD-10	2	8 of 9	8170	93	50	NA	36.45	10.10
Govindarajan	2007	USA,	Cohort	1997–2004	NA	ICD-9	1, 2	7 of 9	87,678	1,371	NA	12.90	NA	NA
Neumann	2012	French,	Cohort	2006–2009	3,125	ICD-10	NA	8 of 9	1,493,472	9,298	69.70	20.70	57.20	26.70
Gu	2013	China	Cohort	2001–2010	4.1	ICD-9, ICD-10	2	9 of 9	8,774	40	NA	NA	NA	51.50
Vallarino	2013	US	cohort	2000–2010	2.2 for pioglitazone 1.9 for insulin	ICD-9	2	8 of 9	56,536	215	68.30	37.40	34.70	31.70
Chang	2012	Taiwan	cohort	2000–2007	NA	ICD-9	2	8 of 9	26,674	5361	80.15	16.87	90.70	36.13
ADOPT	2006	USA, Europe	RCT	2000–2006	4	Adverse event review	2	NA	4351	18	33.40	33.10	33.50	NA
RECORD	2009	Europe, Australia	RCT	2001–2008	5.5	Adverse event review	2	NA	4447	21	75.20%	49.90	74.90	NA

**Abbreviations:** RCT: randomised controlled trials; ICD: International Classification of Diseases; NA: available; ADOPT: A Diabetes Outcome Progression Trial; RECORD: Rosiglitazone Evaluated for Cardiovascular Outcomes and Regulation of Glycaemia in Diabetes.

**Table 2:** Adjustment variables of included observational studies.

Study	HR/OR	Adjustment variables
Smiechowski	Multivariable adjusted	Diabetes duration, HbA1C, obesity, smoking, excessive alcohol use, previous cancer, COPD, asthma, NSAID, aspirin, statins, other antidiabetic drugs
Ruiter	Multivariable adjusted	Age, sex, other medications
Mazzone	Multivariable adjusted	Age, sex, smoking, BMI, HbA1c, medication use
Luo	Multivariable adjusted	Age, ethnicity, education, smoking, BMI, waist-to-hip ratio, recreational physical activity, alcohol intake, total energy intake, percent calories from fat, total fruit/vegetable intake, history of hormone therapy, different treatment assignments for clinical trials
Lai	Multivariable adjusted	Sex, age, pulmonary tuberculosis, COPD, and propensity score
Bodmer	Multivariable adjusted	BMI, smoking, each other
Ferrara	Multivariable adjusted	Age, sex, year of cohort entry, race, income, smoking, glycemic control, diabetes duration, creatinin, congestive heart failure, glucose-lowering drugs
Libby	Multivariable adjusted	Age, sex, smoking, deprivation, BMI, HbA1c, glucose-lowering drugs
Govindarajan	Multivariable adjusted	Age, race/ethnicity, BMI, HbA1C, glucose-lowering drugs
Neumann	Multivariable adjusted	Age, sex, and glucose-lowering drugs
Gu	Multivariable adjusted	Age, sex, smoking, diabetes duration, macrovascular, HbA1c. and glucose lowering agents
Vallarino	Multivariable adjusted	Age, sex, tobacco use, use of medications, medical history
Chang	Multivariable adjusted	Anti-diabetic agents, chronic lung disease, retinopathy, calcium channel blockers, chronic kidney disease, statins, angiotensin receptor blockers, chronic liver disease.

**Abbreviations:** HR: hazard ratio; OR: odds ratio; COPD: chronic obstructive pulmonary disease; NSAID: nonsteroidal antiinflammatory drugs; BMI: body mass index; HbA1C: glycosylated hemoglobin.

### Metformin and lung cancer risk

**Figure 2:**
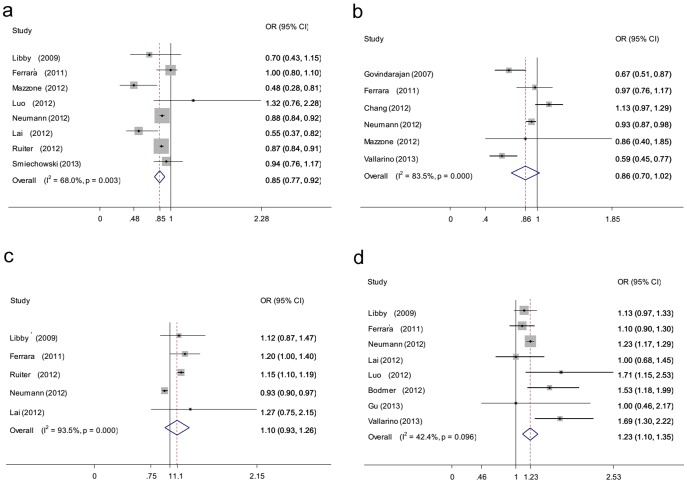
Forest plot of hypoglycaemic agents and the risk of lung cancer in patients with diabetes. a: metformin; b:thiazolidinediones (TZDs); c:sulfonylureas; d:insulin. The squares and horizontal lines correspond to the study-specific OR and 95% CI. The area of the squares reflects the weight (inverse of the variance). The diamond represents the summary OR and 95% CI.

Meta-analysis of all 8 observational studies reported that the metformin use was associated with a statistically significant 15% reduction in lung cancer incidence (OR 0.85, 95% CI 0.77 to 0.92 *P* = 0.003 for heterogeneity) (Figure 2a) [9], [10], [12], [13], [15], [17], [33], [35]. The summary OR of 7 cohort studies was 0.87 (95% CI 0.81 to 0.93). The summary OR of studies that adjusted for other glucose-lowering drugs was 0.87 (95% CI 0.81 to 0.94). A subgroup analysis of seven Western populations showed that metformin exposure was linked to a 13% reduction in lung cancer risk (OR 0.87, 95% CI 0.81 to 0.94). A subgroup analysis was then carried out on only studies that adjusted for smoking. The relation was not statistically significant (OR 0.84, 95% CI 0.61 to 1.06). Separate pooled post-hoc analysis of two RCTs revealed no significant association between metformin and lung cancer (OR 0.65, 95% CI 0.33 to 1.26; *P* = 0.22 for heterogeneity) [27]. The evidence was of low or moderate quality. A subgroup analysis of metformin is shown in table 3.

**Table 3:** A subgroup analysis of metformin use and lung cancer risk in patients with diabetes.

Subgroup analysis	N	OR	95% CI	*P* for heterogeneity	Quality of evidence (GRADE)
RCTs	2	0.65	0.33–1.26	0.22	Moderate
Observational studies	8	0.85	0.77–0.92	0.003	Low
Study design					
Case-control	1	-	-	-	
Cohort	7	0.87	0.81–0.93	0.04	Low
Study location					
Asian	1	-	-	-	
Western	7	0.87	0.81–0.94	0.03	Low
Adjusted for smoking					
Yes	5	0.84	0.61–1.06	0.008	Low
No	3				
Adjusted for glucose-lowering drug					
Yes	7	0.87	0.81–0.94	0.03	Low
No	1	-	-	-	

**Abbreviations:** CI: confidence interval; OR: odds ratio; RCTs, randomised controlled trials.

### TZDs and lung cancer risk

Meta-analysis of 6 observational studies that evaluated the risk of lung cancer with TZDs exposure in patients with diabetes showed that the association was not statistically significant (OR 0.86, 95% CI 0.70 to 1.02; *P* = .00 for heterogeneity) (Figure 2b) [10], [11], [13], [17], [34], [37]. The association was still not significant in the subgroup of five cohort studies (OR 0.86, 95% CI 0.69 to 1.03). However, the result of the Western population showed that TZDs exposure was associated with a 20% reduction in lung cancer risk (OR 0.80, 95% CI 0.62 to 0.98). Separate analysis of two RCTs also did not show an increased or decreased effect of TZDs on lung cancer risk (OR 1.06, 95% CI 0.55 to 2.02; *P* = 0.42 for heterogeneity) [27]. The quality of evidence was ranging from very low to moderate. A subgroup analysis of TZDs is shown in table 4.

**Table 4:** A subgroup analysis of thiazolidinediones use and lung cancer risk in patients with diabetes.

Subgroup analysis	N	OR	95% CI	*P* for heterogeneity	Quality of evidence (GRADE)
RCTs	2	1.06	0.55–2.02	0.42	Moderate
Observational studies	6	0.86	0.70–1.02	0.00	Low
Study design					
Case-control	1	-	-	-	
Cohort	5	0.86	0.69–1.03	0.00	Low
Study location					
Asian	1	-	-	-	
Western	5	0.80	0.62–0.98	0.03	Low
Adjusted for smoking					
Yes	3	0.79	0.47–1.10	0.02	Very low
No	3	0.92	0.72–1.11	0.001	Very low
Adjusted for glucose-lowering drug					
Yes	6	0.86	0.70–1.02	0.00	Low
No	0	-	-	-	

**Abbreviations:** CI: confidence interval; OR: odds ratio; RCTs, randomised controlled trials.

### Sulfonylureas and lung cancer risk

Sulfonylureas use was not associated with lung cancer risk in patients with diabetes (OR 1.10, 95% CI 0.93 to 1.26; *P* = 0.000 for heterogeneity) (Figure 2c) [9], [10], [15], [17], [35]. A subgroup analysis of Western populations pointed out that sulfonylurea use did not modify lung cancer risk (OR 1.09, 95% CI 0.92 to 1.25). Moreover, post-hoc analysis of two RCTs also showed no significant effect of sulfonylureas on lung cancer (OR 1.56, 95% CI 0.75 to 3.26; *P* = 0.57 for heterogeneity) [27]. The evidence was of low or moderate quality. A subgroup analysis of TZDs is shown in table 5.

**Table 5:** A subgroup analysis of sulfonylureas use and lung cancer risk in patients with diabetes.

Subgroup analysis	N	OR	95% CI	*P* for heterogeneity	Quality of evidence (GRADE)
RCTs	2	1.56	0.75–3.26	0.57	Moderate
Observational studies	5	1.10	0.93–1.26	0.000	Low
Study design					
Case-control	0	-	-	-	
Cohort	5	-	-	-	
Study location					
Asian	1	-	-	-	
Western	4	1.09	0.92–1.25	0.000	Low
Adjusted for smoking					
Yes	2	1.18	1.01–1.34	0.66	
No	3	1.06	0.85–1.26	0.000	Low
Adjusted for glucose-lowering drug					Moderate
Yes	4	1.09	0.92–1.25	0.000	Low
No	1	-	-	-	

**Abbreviations:** CI: confidence interval; OR: odds ratio; RCTs, randomised controlled trials.

### Insulin and lung cancer risk

Insulin use was associated with a statistically significant 22% increase in lung cancer risk in patients with diabetes on meta-analysis of eight observational studies (OR 1.23 95% CI 1.10 to 1.35 *P* = 0.096 for heterogeneity) (Figure 2d) [9], [10], [14], [16], [17], [33], [35], [37]. The summary OR of cohort studies was 1.22 (95% CI 1.16 to 1.27). The summary OR of studies that adjusted for other glucose-lowering drugs was 1.22 (95% CI 1.17 to 1.27). Further analysis of the Western population indicated that the OR was 1.26 (95% CI 1.12 to 1.39). The result of studies that adjusted for smoking illustrated that insulin still increased lung cancer risk (OR 1.30, 95% CI 1.09 to 1.51). The evidence was of low or moderate quality. A subgroup analysis of insulin is shown in online table 6.

**Table 6:** A subgroup analysis of insulin use and lung cancer risk in patients with diabetes.

Subgroup analysis	N	OR	95% CI	*P* for heterogeneity	Quality of evidence (GRADE)
RCTs	0	-	-	-	
Observational studies	8	1.23	1.10–1.35	0.096	Moderate
Study design					
Case-control	1	-	-	-	
Cohort	7	1.22	1.16–1.27	0.13	Moderate
Study location					
Asian	2	1.00	0.65–1.35	1	Low
Western	6	1.26	1.12–1.39	0.06	Moderate
Adjusted for smoking					
Yes	6	1.30	1.09–1.51	0.06	Low
No	2	1.23	1.17–1.28	0.28	Moderate
Adjusted for glucose-lowering drug					
Yes	6	1.22	1.17–1.27	0.12	Moderate
No	2	1.26	0.74–1.78	0.06	Low

**Abbreviations:** CI: confidence interval; OR: odds ratio; RCTs, randomised controlled trials.

### Sensitivity analysis

To assess the influence of individual studies on overall outcome, we excluded the studies with the most weight and analyzsed [15] [17]. The conclusions of the main analysis did not change for metformin (OR 0.81, 95% CI 0.67 to 0.95), sulfonylureas (OR 1.09, 95% CI 0.96 to 1.23), TZDs (OR 0.84, 95% CI 0.6 to 1.09) or insulin (OR 1.25, 95% CI 1.06 to 1.44). On replacing this study with similar population-based cohort studies from the same Taiwanese population, where Chang et al. [38] was replaced for sulfonylureas and insulin and where Hsieh et al. [39] was replaced for metformin, there was no significant change in overall association of lung cancer with sulfonylureas (OR 1.05, 95% CI 0.90 to 1.20), insulin (OR 1.36, 95% CI 1.18 to 1.53), or metformin (OR 0.86, 95% CI 0.79 to 0.92).

### Publication bias

The shapes of the funnel plots did not show any obvious asymmetry (Figure S1). The quantitative results of Egger's test still did not suggest the presence of any publication bias (*P* = 0.61 for metformin, *P* = 0.48 for TZDs, *P* = 0.69 for sulfonylureas, and *P* = 0.63 for insulin).

## Discussion

Our comprehensive study quantitatively analysed the possible association between glucose-lowering drugs and incidence of lung cancer in individuals with diabetes. We found that, compared to non-use, metformin use was associated with a 15% decrease in the risk of lung cancer in observational studies, but in the subgroup of studies adjusted for smoking, the protective effect disappeared. Moreover, the preventive effect was not seen in RCTs. Insulin use may be associated with 23% increased lung cancer risk, and the effect persisted in studies adjusted for smoking. Use of TZDs or sulfonylureas did not show an increased or decreased risk of lung cancer.

Metformin is the first-choice glucose-lowering drug in type 2 diabetes. The exact molecular mechanisms connecting metformin use to lung cancer are largely unknown. Metformin inhibits the growth of lung cancer cells and induces apoptosis by activating AMP-activated protein kinase (AMPK), JNK/p38 MAPK signaling pathway [40] [41]. Metformin also exerts an effect by AMPK-independent mechanisms such as inactivation of Raf-ERK-Nrf2 signaling or decreasing plasma IGF-I or receptor tyrosine kinase signaling [4] [42]. Besides a tumor cell-specific effect, metformin has a systemic antiproliferative effect by lowering circulating glucose and insulin levels, contributing to tumorigenesis [43]. Although there is experimental evidence for both cancer treatment and chemoprevention with metformin, clinical chemoprevention is complex and epidemiological studies are inconsistent. Noto et al. [20] recently reported a meta-analysis of metformin and cancer risk, and in their subgroup of lung cancer risk, they observed a 33% lower lung cancer risk with metformin use. However, they included three studies, namely two RCTs and one cohort study, and determined the total risk ratio together. Another meta-analysis demonstrated that metformin use reduces all-cause risk in subjects with type 2 diabetes, but there was no analysis with regard to lung cancer [21]. In our study, we included more studies and also found that metformin was associated with a 15% decreased risk of lung cancer in observational studies, This protective potential of metformin use agrees with previous meta-analysis [20]. Interestingly, in a subgroup of only studies adjusted for smoking, the decrease in risk of lung cancer tended toward null, where this agreed with the meta-analysis of two RCTs. The reason is not clear. A recent study showed that metformin delays the onset of tobacco carcinogen-induced lung tumorigenesis in a non-diabetic mouse model, but the laboratory data are insufficient to translate to humans with diabetes [42].

PPAR-γ is a member of the nuclear receptor superfamily [44]. Once activated, it will preferentially bind to retinoid X receptorα and signal antiproliferative, antiangiogenic, and prodifferentiation pathways in several tissues [5]. In vitro studies have shown that TZDs induce apoptosis and differentiation for potential chemoprevention in non-small-cell lung cancer, but it is not clear whether these mechanisms are relevant in humans [45] [46]. But epidemiological studies have provided varying results, suggesting either a beneficial or neutral effect on lung cancer risk [10] [11] [37]. Colmers et al. [18] observed in their meta-analysis a 9% decreased risk in the lung cancer subgroup among observational studies. Another meta-analysis of Bosetti et al. [19] showed that the TZDs use was not associated with the lung cancer risk, which included two publications for the same population from Taiwan. Our present meta-analysis included one population of the larger sample from Taiwan and another study from the USA [34] [13]. Our updated evidence did not indicate any relevant role of TZDs use on lung cancer risk either in observational studies or in RCTs. However, the association between TZDs and lung cancer risk was pronounced in the Western population when except for a study from Taiwan.

Sulfonylureas seem to promote oncogenesis by increasing insulin secretion, enhancing growth factor-dependent cell proliferation and affecting cell metabolism [8]. Epidemiological evidences in lung cancer are conflicting [10], [17] [15]. A previous meta-analysis suggested that sulfonylurea use significantly increases all-cancer risk in patients with type 2 diabetes [21]. Our overall evidence did not indicate any relevant role of sulfonylurea use in lung cancer risk. The result did not change in the Western population, cohort studies or RCTs. The heterogeneous effects of different sulfonylureas may explain it. Preclinical evidence has also shown that glibenclamide has antitumor activity, besides a role in promoting cancer. The role of glibenclamide as a K_ATP_ channel inhibitor and its interaction with reactive oxygen species(ROS) production seem to underlie the proapoptotic and neoangiogenesis effect [47] [48]. The principal mechanism of action of sulfonylureas on lung cancer has not yet been identified, and clinical data also has shown no association of sulfonylureas with lung cancer. There is a need for further clinical studies according to different sulfonylureas if possible.

Insulin and insulin-like growth factor (IGF) signaling in accelerating neoplastic growth is impressive. Insulin is known to stimulate the proliferation of tumor cells both directly and indirectly by acting upon IGF-1 receptors expressed on lung cancer [49]. Therefore, the use of exogenous insulin may further contribute to neoplastic growth of lung cancer. Increasing epidemiologic evidence suggests insulin effect on both the risk and the prognosis of cancer, but the effect is different in different cancer types. A meta-analysis of reported new use of insulin or insulin glargine was associated with an increased risk of pancreatic cancer, but with a decreased risk of colorectal cancer. They also reported that insulin glargine use had no effect on lung cancer. Comparing non-glargine insulins, two meta-analysis failed to confirm an association between insulin glargine and an increased risk of respiratory tract cancer [50] [51]. Our present analysis focused on ever-used insulin and non-used insulin. Our results indicated that compared with non-insulin use, there was a 23% increased risk of lung cancer in patients with diabetes. These findings were confirmed in subgroup analyses of studies adjusted for smoking or adjusted for other anti-diabetic drugs. Further evaluation of different forms of exogenous insulin is required to better understand this possible association with lung cancer.

The strengths of this study were that we conducted an extensive evaluation of the effects of conventional glucose-lowering drugs on modification of lung cancer risk. Some meta-analyses have shown that metformin use reduces while sulfonylurea and insulin use increases overall cancer risk [21] [20] [52]. However, cancer is a heterogeneous disease, and diabetes differs in the direction and magnitude of relation with site-specific cancer [53]. Thus, the glucose-lowering drug effect on lung cancer risk is necessary. Second, most observational studies are cohort studies and we only included those with adjusted risk estimates controlled for potential confounders such as age, sex, BMI, HbA1C, smoking and so on. A cohort study can provide strong evidence in assessing latent or rare outcomes such as lung cancer incidence [54]. Third, we did a multiple subgroup analysis according to study design, adjusting variables such as smoking and other glucose-lowering drugs. Smoking is the most important risk in lung cancer. We tried to account for this by performing a subgroup analysis restricted to those studies that reported OR after adjusting for smoking. Besides, we also performed a subgroup analysis restricted to those studies that reported OR after adjusting for other glucose-lowering drugs, which may have inherent cancer-modifying effects. Additionally, we conducted a sensitivity analysis and found that removing the studies with the most weight did not have a significant impact on the overall ORs [15] [17]. Finally, With regard to publication bias, both the graphical display of funnel plots and the statistical tests did not indicate any major bias.

There were several limitations in our analysis. First, the meta-analysis was based on data mainly from observational studies because there were only two RCTs. These RCTs were not powerful enough to detect a significant association between glucose-lowering drugs and lung cancer risk, and the subjects included in these studies were not systematically screened for lung cancer, which might have introduced some degree of detection bias. Several observational studies included in our analysis may also have had inherent time-related biases [55]. Second, although we chose the adjusted risk ratio from the original paper, all of the studies did not adjust for the same confounders. Third, the individual studies were limited in reporting an association between glucose-lowering drug and specific pathological type of lung cancer risk. Thus we could not do further analysis according to pathology type. Fourth, evidence quality of meta-analyses in our review was ranging from very low to moderate due largely to a small number of RCTs or heterogeneity. Additionally, the included studies showed heterogeneity. Differences in comparison groups, study population and design, and covariates may explain part of the observed differences between studies. To solve the problem, we did a subgroup analysis to decrease the heterogeneity. Finally, some studies included were population-based and did not specify the type of diabetes. We could not do a subgroup analysis according to type of diabetes. However, over 90% of individuals with diabetes in the general population have type 2 diabetes, so it would have little impact on pooled ORs [56].

In conclusion, based on the results of this meta-analysis, metformin use appeared to be associated with a lower risk of lung cancer in diabetic patients, but the association disappeared when the analysis was restricted to the studies adjusted for smoking. Insulin use increased lung cancer risk, while sulfonylureas and TZDs did not significantly have an association with lung cancer risk. However, this observation needs further investigation before the findings can be translated to clinical practice. A definitive, randomised trial is needed to rigorously assess the effects of glucose-lowering drugs on lung cancer incidence in diabetic patients.

## Supporting Information

Checklist S1 **PRISMA Checklist.**
(DOC)

Figure S1 **Funnel plots of hypoglycaemic agents and the risk of lung cancer in patients with diabetes, a: metformin; b: thiazolidinediones (TZDs); c: sulfonylureas; d: insulin.**
(DOCX)

Table S1 **Risk of bias assessment in Randomised controlled trials.**
(DOCX)
