# Supplementary material for: Conventional Hypoglycaemic Agents and the Risk of Lung Cancer in Patients with Diabetes: A Meta-Analysis
Source: PLoS One. 2014 Jun 12;9(6):e99577. doi: 10.1371/journal.pone.0099577 (PMC4055722; doi:10.1371/journal.pone.0099577)
Supplement: Figure S1 — Funnel plots of hypoglycaemic agents and the risk of lung cancer in patients with diabetes, a: metformin; b: thiazolidinediones (TZDs); c: sulfonylureas; d: insulin. (DOCX) [file pone.0099577.s002.docx]

**
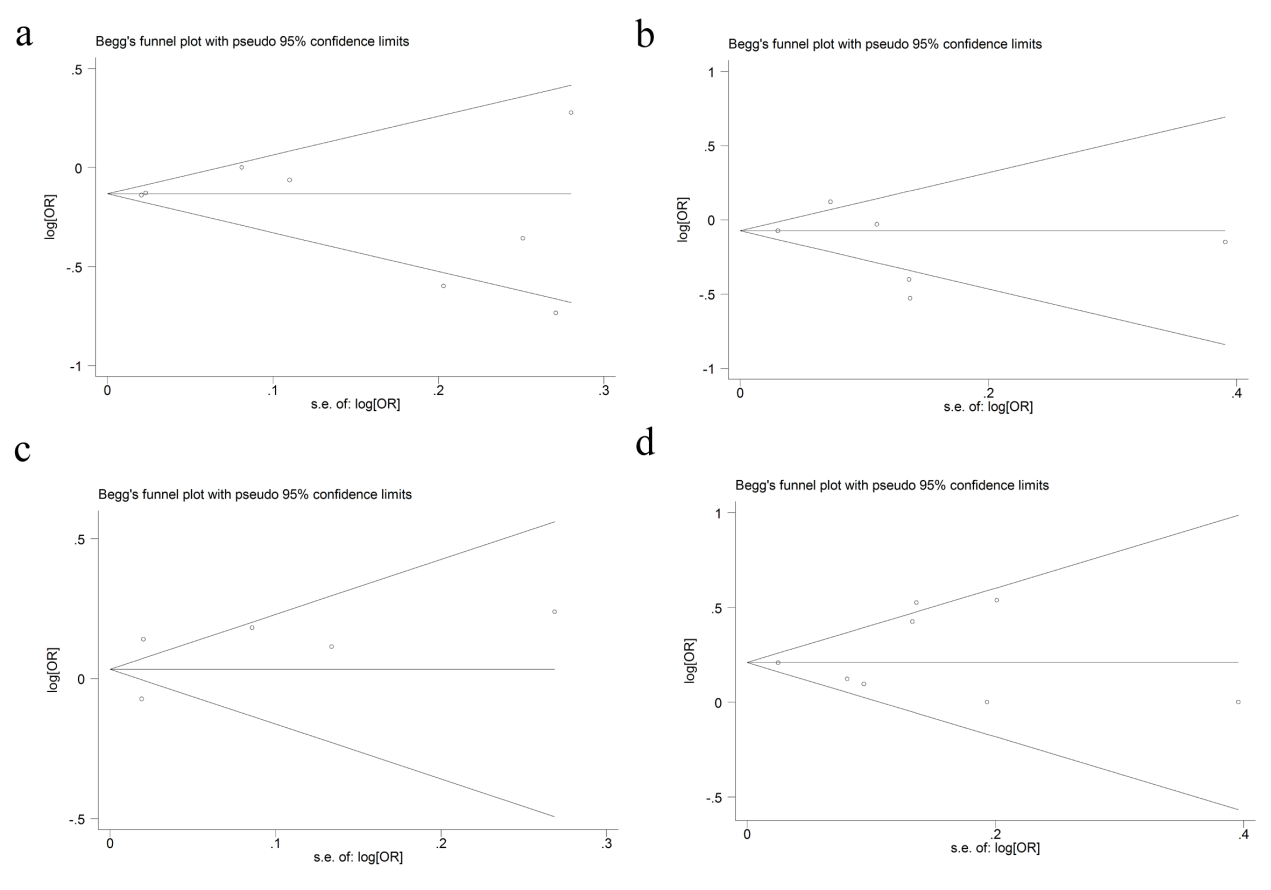
Figure S1.** **Funnel plots of hypoglycaemic agents and the risk of lung cancer in patients with diabetes,** a: metformin; b: thiazolidinediones (TZDs); c: sulfonylureas; d: insulin.
