# Supplementary material for: Conventional Hypoglycaemic Agents and the Risk of Lung Cancer in Patients with Diabetes: A Meta-Analysis
Source: PLoS One. 2014 Jun 12;9(6):e99577. doi: 10.1371/journal.pone.0099577 (PMC4055722; doi:10.1371/journal.pone.0099577)
Supplement: Table S1 — Risk of bias assessment in Randomised controlled trials. (DOCX) [file pone.0099577.s003.docx]

TableS1

Risk of bias assessment in Randomised controlled trials

|  | Selection bias | Performance bias | Detection bias | Attrition bias | Reporting bias |
| --- | --- | --- | --- | --- | --- |
| ADOPT | A | A | A | C | B |
| RECORD | A | A | A | C | B |

A: Low risk of bias B: Unclear risk of bias C: High risk of bias
